# Pancreaticoduodenectomy following total occlusion of the superior mesenteric artery: a case report and literature review

**DOI:** 10.1186/s40792-019-0718-2

**Published:** 2019-11-04

**Authors:** Reo Ohtsuka, Hodaka Amano, Michiyo Hashimoto, Toshiyasu Iwao

**Affiliations:** 1 Department of Surgery, Aidu Chuo Hospital, 1-1 Tsuruga-machi, Aizuwakamatsu, 965-0011 Japan; 2 Department of Gastroenterology, Aidu Chuo Hospital, 1-1 Tsuruga-machi, Aizuwakamatsu, 965-0011 Japan

**Keywords:** Cholangiocarcinoma, Pancreaticoduodenectomy, Superior mesenteric artery occlusion, Celiac artery occlusion

## Abstract

**Background:**

Patients with chronic occlusion of the celiac artery and superior mesenteric artery (SMA) are often asymptomatic, and occlusion may be caused by arteriosclerosis or median arcuate ligament compression. Pancreaticoduodenectomy (PD) is occasionally performed for patients with celiac artery occlusion; however, reports on patients with SMA occlusion are rare. We report a patient with cholangiocarcinoma and total atherosclerotic occlusion of the SMA without preoperative stenting or bypass.

**Case presentation:**

A 73-year-old man suspected to have lower bile duct carcinoma was admitted to our hospital for further treatment. Three-dimensional computed tomography (3DCT) showed a common bile duct tumor and total occlusion of the SMA with collateral circulation of the gastroduodenal artery (GDA) and inferior mesenteric artery (IMA). We performed a PD. During the operation, we used test clamping of the GDA, which revealed no bowel ischemia. The postoperative course was uneventful, and the patient was discharged on postoperative day (POD) 30. 3DCT on POD 98 and POD 307 showed development of collateral circulation between the IMA and SMA.

**Conclusion:**

Here, we report the case of a patient with total occlusion of the SMA who subsequently underwent PD. 3DCT was instrumental in gathering vascular collateral information and thus we conclude that the assessment of collateral circulation before surgery is important.

## Background

Patients with chronic occlusion of the celiac artery (CA) and superior mesenteric artery (SMA) are often asymptomatic and occlusion may be caused by arteriosclerosis or median arcuate ligament compression [1]. In chronic obstruction, the intestinal blood flow is maintained by the formation of collateral circulation. Pancreaticoduodenectomy (PD) is occasionally performed for patients with CA occlusion; however, there are few reports of patients with SMA occlusion. Stenting is used proactively in patients with SMA and CA occlusion [2]. We report the case of a patient with cholangiocarcinoma and total atherosclerotic occlusion of the SMA without preoperative stenting or bypass.

## Case presentation

**Fig. 1:**
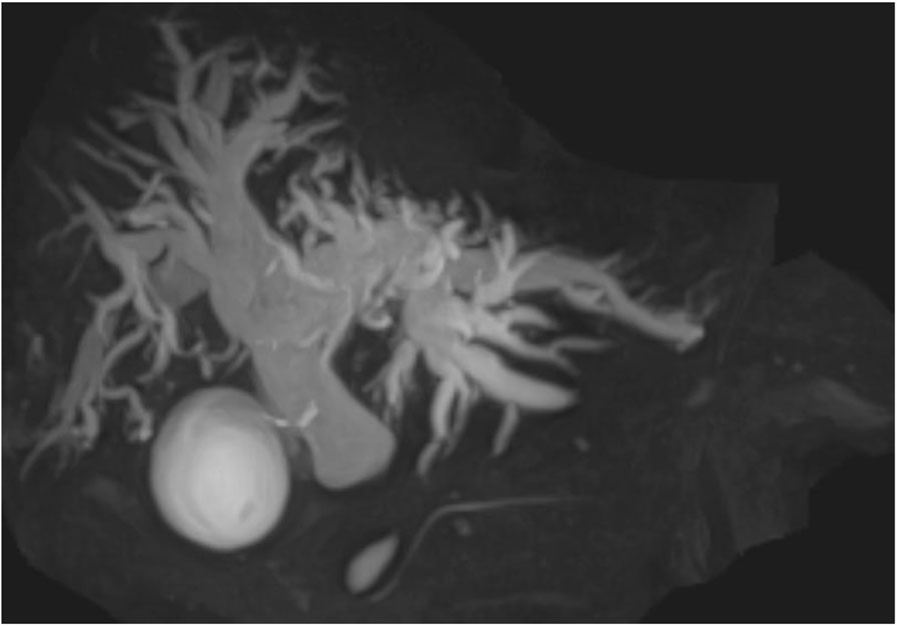
Magnetic resonance cholangiopancreatography showing stenosis of the lower bile duct

**Fig. 2:**
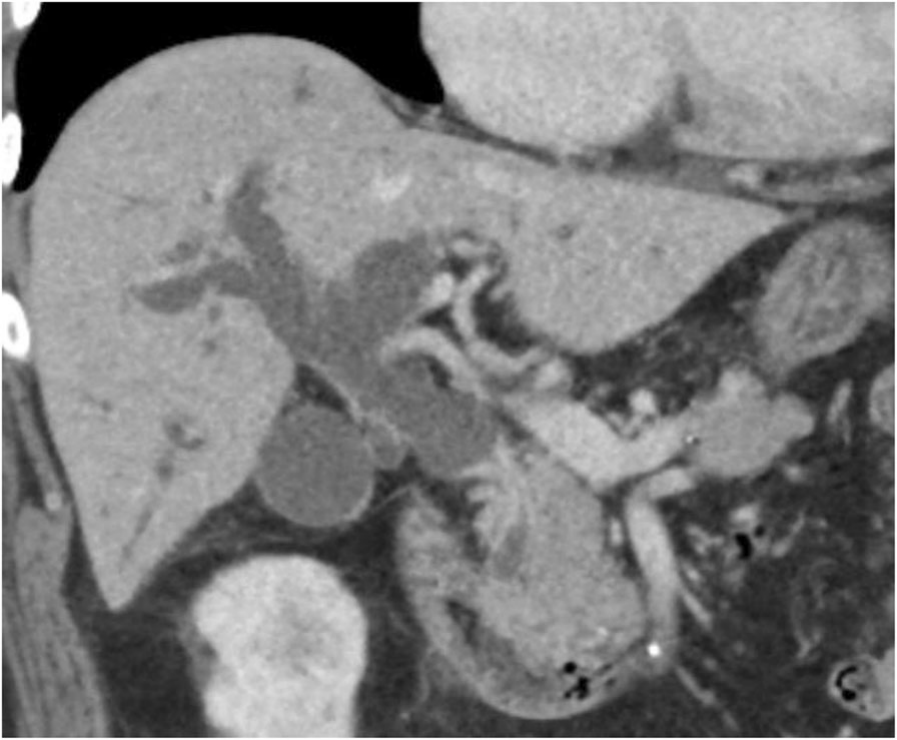
Three-dimensional computed tomography showing a common bile duct tumor

**Fig. 3:**
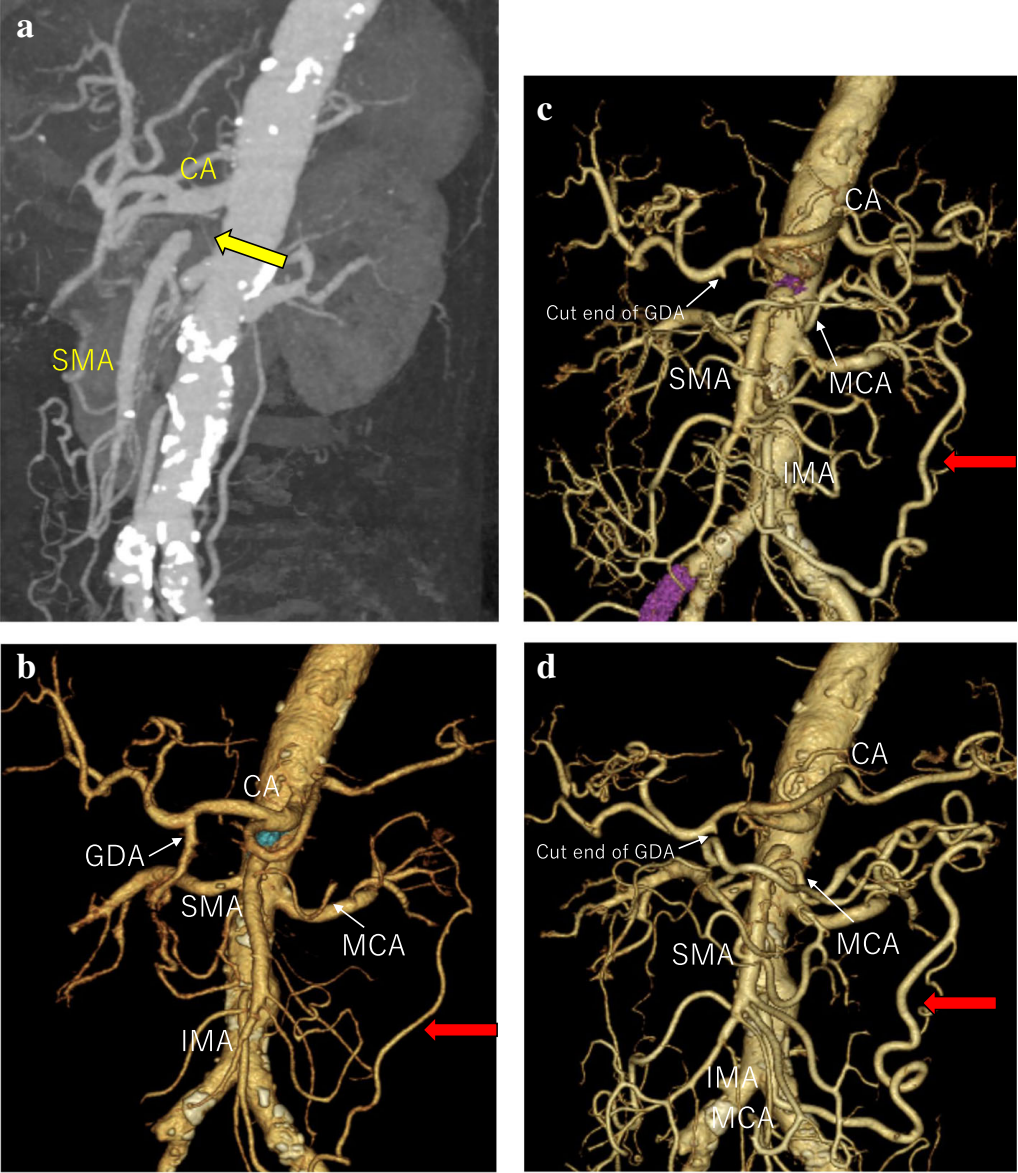
Superior mesenteric artery occlusion and SMA-IMA anastomosis. **a** Superior mesenteric artery occlusion was seen before surgery (yellow arrow). **b** Dilation of SMA and IMA branches before surgery. **c** Arc of Riolan development observed on postoperative day 98. **d** Arc of Riolan development observed on postoperative day 307

A 73-year-old man suspected of having cancer of the lower bile duct was admitted to our department for further treatment. The patient had hypertension, type 2 diabetes mellitus (DM), and atrial fibrillation. Laboratory findings revealed elevation of total and direct bilirubin as well as alkaline phosphatase. Endoscopic retrograde cholangiopancreatography and magnetic resonance cholangiopancreatography showed stenosis of the lower bile duct, which strongly suggested the presence of cholangiocarcinoma (Fig. 1). Three-dimensional computed tomography (3DCT) showed a common bile duct tumor and total occlusion of the SMA with collateral circulation of the gastroduodenal artery (GDA) and inferior mesenteric artery (IMA) (Figs. 2 and 3a, b).

We performed a PD with regional lymphadenectomy on the patient. Intraoperatively, test clamping of the GDA was performed. Before and after the GDA clamp, no change was observed in the intestinal color tone or the beating of blood vessels (from the SMA to the blood vessels in the mesenteric periphery). Therefore, resection of the GDA was performed. Dissection of the mesopancreas was performed over the superior mesenteric vein, followed by dissection of the common bile duct. Frozen section evaluation showed negative margins. The final histology revealed a 10 × 12-mm-sized poorly differentiated adenocarcinoma of the bile duct. There was no lymph node metastasis, but vascular and perineural infiltration was confirmed. There was an International Study Group of postoperative Pancreatic Fistula, grade A pancreatic fistula, but the course was uncomplicated and S-1 chemotherapy was started from postoperative day (POD) 27. The patient was discharged on POD 30. He received 3 months of adjuvant S-1 chemotherapy, which was eventually discontinued because of eczema and dysgeusia. 3DCT on POD 98 and POD 307 showed development of Arc of Riolan between the IMA and SMA (Fig. 3c, d).

According to Gaujoux et al., 27 (5%) of the 545 patients who underwent PD had hemodynamically significant stenosis in the CA or SMA with multidetector CT [1]. Most cases of SMA stenosis and occlusion were due to atherosclerosis; SMA stenting or bypass was performed for these patients [2]. Acute SMA occlusion typically presents with massive mesenteric ischemia, but in chronic occlusion, collateral formation between the GDA and IMA preserves mesenteric blood flow. Although there have been several studies on the natural history of asymptomatic CA/SMA stenosis, it is rare that symptoms due to ischemia occur if all three arteries (CA, SMA, and IMA) do not have severe stenosis [3]. van Petersen analyzed 672 cases of chronic stenosis with CA/SMA over a period of 8 years and classified the collaterals of mesenteric arteries as the gastroduodenal, arc of Buhler, Riolan, and Drummond, and he described the narrowing of the blood vessels and the development of collateral pathways [4]. When performing PD in a patient with chronic stenosis of the SMA, it is necessary to remove the GDA. Because of the concern about intestinal ischemia, it is necessary to recognize the hemodynamics of the collateral pathways in advance. Although many cases of PD for CA occlusion have been reported, only ten cases, including our case, have been reported in patients with benign SMA occlusion who have undergone PD or total pancreatectomy (Table 1). In many cases, the underlying diseases include hypertension and hyperlipidemia, which are considered to be caused by arteriosclerosis. As a response at the time of surgery, patients in whom stents were applied to the SMA before surgery and patients in whom blood flow was evaluated by a clamp test of the GDA to confirm the absence of ischemia followed by GDA resection have been reported. A summary of the collateral circulation is found in Table 1, and Riolan or Drummond circulation developed in all cases. In surgery for pancreatic cancer, colonic resection may be performed because of colon infiltration, but, in the case of SMA occlusion, resection of the collateral circulation via the IMA to middle colic artery is also required. In such cases, a bypass or stenting with the SMA is required. We were preparing for SMA reconstruction. The cause of SMA occlusion in this case may be attributed to arteriosclerosis because hypertension and DM were the underlying diseases. In this patient, we evaluated the SMA occlusion and collateral circulation with preoperative 3DCT and confirmed that there was a collateral formation from the IMA, following which PD was performed. Here, we evaluated the SMA occlusion and collateral circulation with preoperative 3DCT; collateral circulation from the IMA was confirmed and we performed PD. It is useful to examine the presence or absence of intestinal ischemia by clamping the GDA intraoperatively with angiotribe forceps, and PD could be performed without SMA reconstruction. In our case, the development of the Arc of Riolan or Drummond, which are arcade pathways, was able to be gradually confirmed by 3DCT on POD 98 and POD 307 after the surgery (Fig. 3; Table 1).

**Table 1:** Ten cases of patients with benign SMA occlusion who have undergone PD or total pancreatectomy

Reference	Age, sex	Diagnosis	Operative procedure	Site of occlusion	Past history	Communication	Etiology of occlusion	Treatment for occlusion
[1]-1	63	IPMN	PD	SMA	/	/	Arteriosclerosis	Preoperative SMA stenting
[1]-2	68	PC	PD	SMA	/	/	Arteriosclerosis	Preoperative SMA stenting
[1]-3	68	PC	PD	SMA	/	/	Arteriosclerosis	Postoperative diagnosis, bowel resection
[5]	64, F	PC	PD	CA and SMA	HT, DM	IMA-SMA	Arteriosclerosis	Heparinization
[6]	60, M	PC	PD	CA and SMA	HT	IMA-SMA		
[7]	67, F	PC	PD	SMA	RA	IMA-SMA	Arteriosclerosis	Measurement of blood flow
[8]	69, M	PC	PD	CA and SMA	HL	IMA-SMA	Arteriosclerosis	
[9]	69, F	PC	PD	SMA	HL		Arteriosclerosis	Thrombectomy
[10]	58, M	PC	Total pancreatectomy	SMA		Dilatation of posterior and anterior pancreaticoduodenal arcades.	Arteriosclerosis	Preoperative SMA stenting
Our case	73, M	CC	PD	SMA	HT, DM	IMA-SMA	Arteriosclerosis	Clamp test

*IPMN* intraductal papillary mucinous neoplasm, *PC* pancreatic cancer, *CC* cholangiocarcinoma, *PD* pancreaticoduodenectomy, *CA* celiac artery, *SMA* superior mesenteric artery, *IMA* inferior mesenteric artery, *HT* hypertension, *DM* diabetes mellitus, *RA* rheumatoid arthritis, *HL* hyperlipidemia

## Conclusions

We describe a patient with total occlusion of the SMA who subsequently underwent PD. 3DCT is useful to examine vascular collateral circulation as the assessment of the collateral circulation before surgery is important. To our knowledge, this is the first case with observation of the development of collateral circulation between the SMA and IMA in the postoperative course after PD following total occlusion of the SMA.

## Data Availability

The datasets supporting the conclusions of this article are included within the article and its additional files.

## Abbreviations

3DCT: Three-dimensional computed tomography
CA: Celiac artery
CT: Computed tomography
DM: Diabetes mellitus
GDA: Gastroduodenal artery
IMA: Inferior mesenteric artery
PD: Pancreaticoduodenectomy;
POD: Postoperative day
SMA: Superior mesenteric artery
